# Genetically inherited tolerance may unveil trait dominance patterns in an amphibian model

**DOI:** 10.1038/s41598-019-55838-9

**Published:** 2019-12-16

**Authors:** E. Fasola, R. Ribeiro, I. Lopes

**Affiliations:** 1 Department of Biology & CESAM (Centro de Estudos do Ambiente e do Mar), University of Aveiro, Campus de Santiago, 3810-193 Aveiro, Portugal; 2 CFE – Centre for Functional Ecology, Department of Life Sciences, University of Coimbra, Calçada Martim de Freitas, 3000-456 Coimbra, Portugal

**Keywords:** Evolutionary ecology, Evolutionary biology

## Abstract

Chemical contamination may cause genetic erosion in natural populations by wiping out the most sensitive genotypes. This is of upmost concern if the loss of genetic variability is irreversible due to contaminant-driven elimination of alleles, which may happen if tolerance is a recessive or incompletely dominant trait – the recessive tolerance inheritance (working-) hypothesis. Accordingly, this work investigated the tolerance inheritance to lethal levels of a metal-rich acid mine drainage (AMD) and to copper sulphate in a population of *Pelophylax perezi*. Time-to-death for each egg, after being exposed to 60% of a sample of acid mine drainage and to 9 mg/L Cu, was registered, and, for each egg mass, the median lethal time (LT_50_) and respective quartiles (LT_25_ and LT_75_) were computed. Results suggested that genetically determined tolerance could be probably driven by incomplete dominance (with possible maternal effect influence), preliminarily supporting the initial hypothesis.

## Introduction

Understanding the tolerance of natural populations to stressors is important for conservation. Acting as a directional selective pressure, a chemical stress can result in genetic erosion^1^, mainly if the within- (sensitive) genotype trait variability is low^2,3^. Researchers discussed if genetically determined tolerance stems from few major genes or from many minor genes (each giving a small contribution to the final phenotype). Both cases have been reported and were related with the intensity of the selective pressure^4–6^. Under mild contaminant concentrations, tolerance seems to have a polygenic basis, while under intense pollution pulses only few genes seem to play a central role^5–7^. Adaptation should normally be achieved by the spread of many genes, each of small effect (polygenic determination), and adaptation by major genes should be unusual^5–7^. However, the response to contaminants has frequently resulted from the spread of major genes^5,6,8–11^. An example is that of the tolerance to DDT, in the common house fly, *Musca domestica*^11,12^; as well as in the *Anopheles gambiae* complex^8^. This is valid for other insecticides^13^, such as the case of the rusty grain beetle, in which the tolerance to phosphine is inherited as an incompletely dominant trait^10^. However, there are some dominant factors that comes into play only at high pesticide concentrations^10^. Tolerance to a rodenticide was determined by a single gene^14,15^. The same was true for tolerance to dioxin-like compounds in fish^16,17^ and to trace metal in plants and animals^4–7,18^.

The Strategic Plan for Biodiversity 2011–2020 highlights the importance to preserve genetic diversity to minimize “genetic erosion”^19^. Contaminant-driven genetic erosion has been reported resulting from genetic drift bottlenecks and from natural directional selection^1–3,20^. Genetic erosion can act directly, by eradicating the most sensitive genotypes and/or, indirectly, by altering among-species interactions (e.g., patterns of predation or competition). The irreversible loss of alleles is one of its most serious possible consequences, unless gene flow or mutations counterbalance it afterwards^3^. Another consequence is the decrease of population growth rate and ultimately population extinction due to a reduction in the average fitness^2,3^. Small populations of species with limited mobility (thus failing to ensure gene flow) are, possibly, the most threatened by genetic erosion. Amphibians fit this description and, moreover, are facing a global decline^21,22^.

The present work aimed at testing the recessive tolerance inheritance (working-) hypothesis^3^: “A contaminant [acting as a directional selection pressure] can eliminate alleles if [the genetic component of] tolerance is a recessive or incompletely dominant [including incomplete recessivity] trait”. Under mild stressful conditions, fitness tends to correlate with heterozygosity^23–25^. For example, higher proportions of heterozygous genotypes in *Gambusia affinis*, *Pimephales notatus* and *Fundulus notatus* were reported in populations of a river receiving acid mine drainage, when compared with reference populations^26^. Therefore, overdominance — heterozygotes being more tolerant than either homozygotes — would be expected to be the rule of tolerance inheritance. This is because heterozygotes frequently present a higher metabolic efficiency than homozygotes^27^. However, the dominance level can change with the stressor intensity^5,7,27^. Therefore, the tested hypothesis is plausible following exposure to almost fully lethal levels of contaminants, which is expected to have a strong demographic impact in the population. If it would be confirmed, the loss of alleles is most probable, especially if full recessivity is found instead of incomplete dominance. Incomplete dominance refers to all situations where the individual phenotype is not fully polymorphic (being either sensitive or tolerant), instead following a gradient depending on the degree of dominance (each individual being either sensitive or tolerant or in-between) of the trait (tolerance)^5^.

In the present study, tolerance inheritance was investigated by observing the frequency of organisms with different levels of tolerance (selected trait) subjected to a strong directional selection (i.e., a selectable marker). Observations were made in Iberian Water Frog, *Pelophylax perezi*, egg masses, comparing the observed patterns of tolerance inheritance within egg masses with the expected distributions (dominant, recessive, underdominant, overdominant, and incompletely dominant) (see Materials and Methods, paragraph 2.5 and Fig. S1 in Supplementary Materials). This was done by: (i) investigating whether populations exhibit “critically sensitive genotypes”^3^ and (ii) comparing among and within egg masses variability in time to death. If genetically determined tolerance corresponds to a recessive allele, then the most tolerant and the most sensitive egg masses should present lower variability compared with masses showing intermediate median lethal time values. The worst-case scenario of the recessive tolerance inheritance hypothesis would correspond to an inverted U-shaped relationship between the within egg mass variability and the allele(s) determining sensitivity. In the present work, only the simplest system of genetic determination – one gene with two alleles – was fully discussed because the same rationale on the probability of losing recessive alleles applies to more complex scenarios – one gene with more than two alleles and/or two or more genes – which involve an overly high number of combinations.

In the present study, acid mine drainage (AMD), and copper were chosen as test contaminants because the test species is known to inhabit ponds contaminated by small dilutions of AMD^28^ and copper is one of its key components^29^. Furthermore, correlations between toxicity of AMD and copper have been previously found in invertebrates^30^.

## Methods

### Study organism

The model organism for this study was the Iberian Water Frog: *Pelophylax perezi* (López-Seoane, 1885). The species has a conservation status of least concern^31^. It is endemic and common in the Iberian Peninsula^31–33^. It can colonize eutrophic and even contaminated waters^32,33^. The ideal approach to address tolerance inheritance would be to decide all crossings under fully controlled conditions. However, rearing *P. perezi* in the laboratory is very difficult because mortality of the adults can be high and the efficiency of reproduction is reduced (personal observation). Comparatively, to study egg masses collected in the field was a much weaker approach, namely because the occurrence of maternal effects cannot be ruled out. However, this was the only viable approach to preliminarily tackle our objectives.

The authorisation for egg sampling was approved by the competent national authority: The Institute for Nature Conservation and Forests, ICNF (Number 394). Laboratory tests with eggs are not considered to be tests with vertebrates according to the European directive EU2010/63 on the protection of animals used for scientific porpoises. All the practices conducted in our laboratory are approved by the competent national authority: Direcção-Geral de Alimentação e Veterinária (DGAV).

### Assay setup - AMD

Twenty one *P. perezi* egg masses were collected, from April 2013 to August 2013, at Quinta da Boa Vista (40°35′48″N–8°41′43″W), which is considered a non-contaminated reference site. Only masses containing eggs at developmental Gosner’s stages 8 to 10 were collected. In the laboratory the masses were housed in FETAX medium under matching environmental conditions and tests started in the same day of field collection. Eggs were exposed to a 60% dilution (with FETAX) of the acid mine drainage (AMD) collected at the São Domingos mine (37°39′15″N–7°30′31″W), this AMD is very acid (pH ≈ 2) and rich in metals^29^. Metal concentrations were quantified in the 100% AMD and in a 10% dilution (see Supplementary Materials and Table T1).

Eggs from different egg masses were not mixed. For each egg mass, control treatment (FETAX medium) was performed in four replicates (with five eggs each, to control for the health status of the eggs and validate the toxicity assay)^34^; while the number of eggs (and replicates) exposed to the test solution varied depending on the mass size (usually between 80 and 120 eggs). The eggs’ jelly coat was not removed to mimic natural scenarios. Tests were performed at 23 °C under a 14 h/10 h light/dark photoperiod. Embryos were checked for death accordingly to Dawson and Bantle^35^ (at a 10x magnification using a Leica MS5 microscope) following a predetermined base 10 logarithmic time scale (by successively adding 0.15 to the log_10_720): 12 h 00 min (=720 min), 16 h 57 min, 23 h 57 min, 33 h 49 min, 47 h 46 min, 67 h 29 min, and 95 h 19 min. Therefore, the exposure lasted about 96 h (see Supplementary Materials).

### Assay setup - copper

Sampling was performed from March 2014 to May 2014 and from March 2016 to April 2016. The toxicity tests were performed as above, after sampling 40 egg masses (20 per sampling season). The test solution, was made by diluting, with FETAX medium, a stock solution (100 mg/L Cu) of CuSO_4_•5H_2_O, until a 9 mg/L of Cu concentration (resulting pH ≈ 7). This value was intended to be lethal within a 96 h exposure period (see Supplementary Materials).

### Statistical analysis

AMD-exposed egg masses were labelled with alphabet letters from A to U; copper-exposed masses were labelled from A14 to T14 and A16 to T16 (in order from the most sensitive to the most tolerant, i.e. increasing tolerance). Within each mass, eggs were classified from extremely sensitive to extremely tolerant depending on when they were found dead during the experiment, from 12h00m up to more than 95h19min. Median, lower and upper quartiles of lethal time values (LT_50_, LT_25_ and LT_75_, respectively) of each egg mass were calculated using the PriProbit software^36^. An egg mass was categorized as critically sensitive when its LT_75_ was below the median of the set of LT_50_ values and as safely tolerant when its LT_25_ was above the median LT_50_^3^. The within egg mass variability in time to death responses was calculated as the relative spread, which is the interquartile distance – LT_75_ minus LT_25_ – divided by its median – the LT_50_ for each egg mass^3^. The remaining analyses were performed using Statistica for Windows 8.0 (StatSoft, Tulsa, OK, USA) and IBM SPSS for Windows v24 (IBM Corporation, Armonk, NY, USA), please see Supplementary Materials. Median lethal time values (LT_50_) were checked for parametric correlation (Spearman test) versus the respective relative spread values. Comparison between LT_50_ and spread values resulting from the two sampling seasons was performed conducting a Mann-Whitney U-test.

### Inheritance patterns evaluation

To investigate tolerance inheritance (as a dominant, recessive, overdominant, underdominant, or incompletely dominant trait), graphs were prepared showing the expected patterns of F1 tolerance results for the simplest possible scenario: tolerance being a trait determined by a single gene with only two alleles (Fig. S1). Because the patterns were evaluated by their displayed time to death frequencies, the classification was made taking into account the expected pattern for each scenario (dominant, recessive, overdominant, underdominant, or incompletely dominant trait). For example, tolerance as a dominant trait (in homozygous dominant and heterozygous individuals) would result eggs surviving until the end of the assay, thus a right skewed pattern (see Supplementary Materials, Fig. S1, for further details).

## Results

Neither field environmental parameters, at eggs collection days (rank along the breeding season, average air temperature, amount of rain, average wind speed, and average insulation), nor pH and conductivity during laboratory testing correlated with LT_50_ values (p > 0.05; values of r: 0.39, 0.40, 0.42, 0.32, 0.31, 0.32, and 0.33, respectively).

### AMD assays

**Figure 1:**
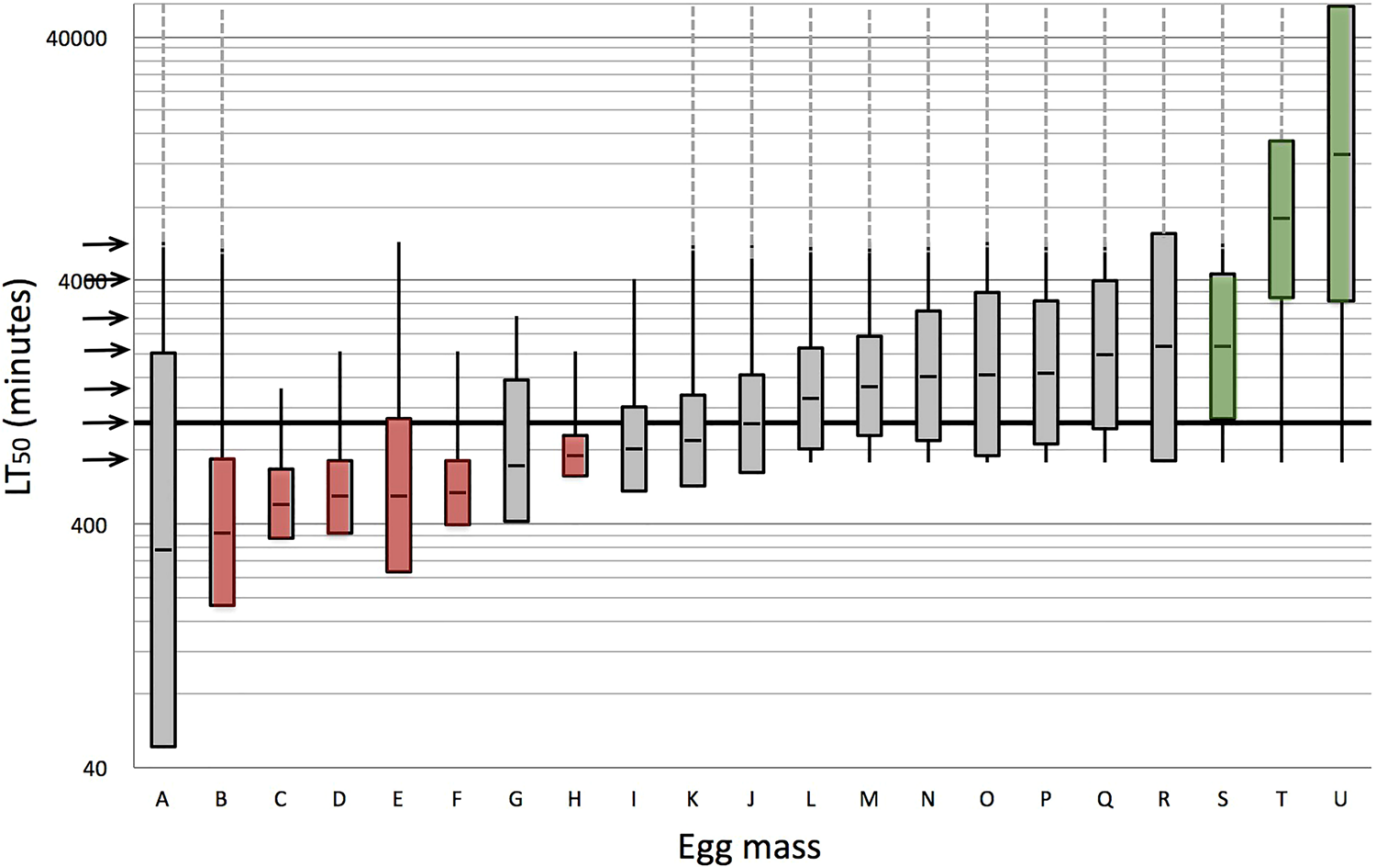
Box plots representing the median, the lower and the upper quartiles of lethal time values (exposure times after which 50, 25 and 75% of eggs died) of eggs within each of 21 Iberian Water Frog egg masses (**A–U**) exposed to a 60% dilution of acid mine drainage. Vertical black lines represent maximum and minimum lethal time values for each egg mass (until the last observation made). Grey vertical dashed lines indicate some eggs were still alive at the end of the last observation. Grey solid lines correspond to the scale of the graph, indicating the scale’s intervals. The thick horizontal black line represents the average of the 21 median lethal time values. Observations, indicated by horizontal arrows, were made 720, 1017, 1436, 2029, 2866, 4048, and 5719 minutes after the start of the assay. Red and green box plots represent egg masses identified as critically sensitive and safely tolerant, respectively.

A broad range of egg mass tolerance to AMD, from critically sensitive (masses from B to F and H) to safely tolerant (T and U) was recorded (Table T2, Figs. 1 and S2). All egg masses had relative spread values below 300% except for masses A and U (the most sensitive and the most tolerant, respectively) (Table T2 and Fig. S3). The expected inverted U-shaped relationship between relative spread and LT_50_ values was not found due to the most extreme egg masses – A and U – for which LT_50_ values were severely extrapolated. When excluding them, a statistically significant model fit corresponding approximately to the expected shape was obtained (p < 0.001) (Fig. S3).

### Copper assays

**Figure 2:**
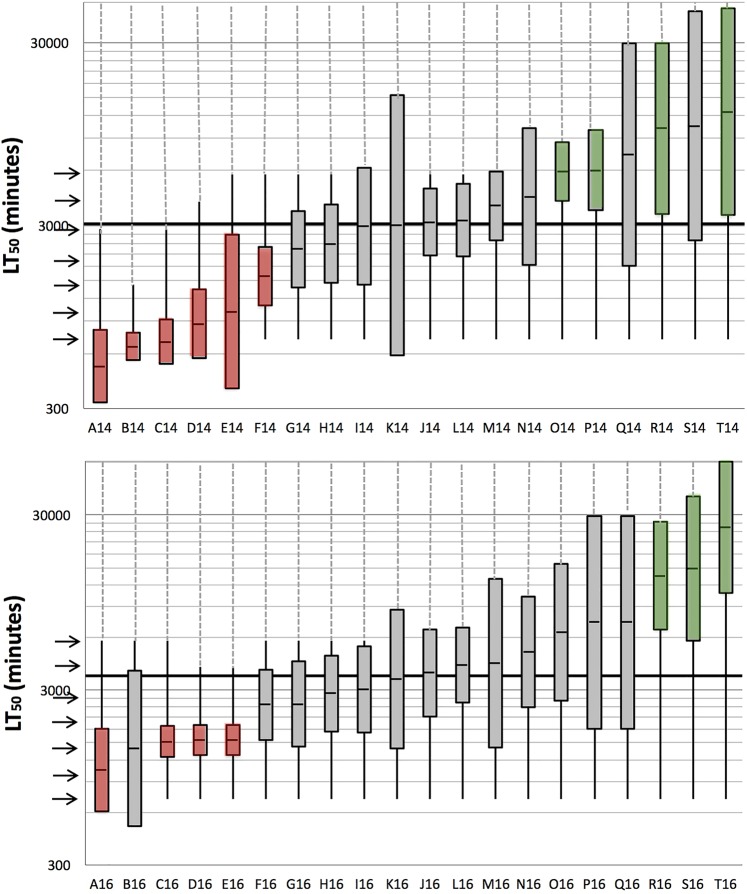
Box plots (2014 sampling above, 2016 sampling below) representing the median, the lower and the upper quartiles of lethal time values (exposure times after which 50, 25 and 75% of eggs died) of eggs within each of 20 Iberian Water Frog egg masses (A14 to T14 from 2014 and A16 to T16 from, 2016), exposed to 9 mg/L of copper. Vertical black lines represent maximum and minimum lethal time values for each egg mass (until the last observation made). Grey dashed vertical lines indicate some eggs were still alive at the end of the last observation. Grey solid lines correspond to the scale of the graph, indicating the scale’s intervals. The thick horizontal black line represents the average of the 20 median lethal time values. Observations, indicated by horizontal arrows, were made 720, 1017, 1436, 2029, 2866, 4048, and 5719 minutes after the start of the assay. Red and green box plots represent egg masses identified as critically sensitive and safely tolerant, respectively.

Even larger ranges of egg mass tolerance were found in both experiments with copper (Tables T3 and T4). Masses from A14 to F14 were found to be critically sensitive, but no safely tolerant egg masses were registered (Fig. S4). Egg masses E14, I14, and K14 showed clear bimodal distribution patterns (Fig. S4). All egg masses had relative spread values below 300% with four exceptions: masses K14, Q14, S14, and T14 (being S14 and T14 most tolerant egg masses) (Table T3 and Fig. S5). In the second copper assay, egg masses A16, and C16 to E16 were found to be critically sensitive, while egg masses from R16 to T16 were safely tolerant (Table T4 and Figs. 2 and S4). The egg mass B16 was the only ones presenting a bimodal distribution (Fig. S4). All egg masses had relative spread values below 300% with two exceptions: masses P16 and Q16 (Table T4 and Fig. S5). No significant differences were found between 2014 and 2016 sampling seasons (p > 0.05) (Tables T3 and T4). In contrast with AMD, an inverted U-shaped relationship between relative spread and LT_50_ values was not found (Fig. S5).

## Discussion

Genetic determination (with the possibility of epigenetics^37^;) and stochasticity were probably the most responsible factors explaining the registered variability in tolerance, because neither trends within breeding seasons nor correlations with any field or laboratory environmental parameter were found. However, this lack of patterns did not fully exclude the occurrence of phenotypic plasticity and maternal effects, which occur commonly in some species of amphibians^2,38^. The embryos of some species can express such maternal effects, depending on the size of the yolk in the eggs; eggs with larger yolk would hatch larger larvae^38^. It is documented that female frogs show variability in egg’s yolk size, for different egg masses, but only for populations inhabiting very environmental unstable environments^38,39^; which was not the case of the reference site visited in the present work.

The aim of this work was to test the recessive tolerance inheritance (working-) hypothesis. To support its worst-case scenario – (full) recessivity – each of the tested egg masses should fall in one of the following categories: (i) all eggs being highly sensitive, with small relative spread (at least one parent being homozygous dominant); approximately matched by egg masses C, D, F, and H for AMD and by egg masse B14 for copper; (ii) all eggs being highly tolerant, with small relative spread (both parents being homozygous recessive); matched by no egg mass (Figs. S1, S2, S4); (iii) eggs, within each egg mass, being either highly tolerant or highly sensitive in similar (~50%) proportions, with very large relative spread (the crossing of a heterozygous with a homozygous recessive frog); matched by no egg mass for AMD and approximately matched by egg masse K14 for copper; (iv) similar to the previous category but with a much higher proportion (~80%) of highly sensitive eggs, with large relative spread (both parents being heterozygous); approximately matched by egg mass A, E and G for AMD and mass E14 for copper (Figs. S1, S2, S4). The first and second categories are also possible outcomes of all other tolerance inheritance patterns (dominance, overdominance, underdominance, and incomplete dominance). The third category may also be a possible outcome of all patterns except the incomplete dominance.

Only six out of 21 egg masses exposed to AMD and only three out of 40 egg masses exposed to copper were found to possibly support the (full) recessivity mechanism for tolerance. Only egg masses matching the fourth category (3 out of 21 egg masses exposed to AMD and one out of 40 egg masses exposed to copper) fully support tolerance being recessive because this category is a possible outcome of no other inheritance pattern.

If tolerance is inherited as a (fully) dominant, overdominant or underdominant trait, then neither a partially lethal nor even an almost fully lethal input of AMD or copper would eliminate alleles from the impacted population, since either the heterozygote would be maximally tolerant (dominance and overdominance) or both homozygotes were the most tolerant individuals (underdominance). In the AMD assay, eight (egg masses A, C, D, E, F, G, H, T, and U) out of 21 (38%) egg masses could support (full) dominance, overdominance or underdominance (Figs. S1 and S2). Whereas in the copper assays, nine (B14, E14, K14, P14, R14, R16, T14, Q16, R16, and T16) out of 40 (22.05%) egg masses could match those pattern of tolerance inheritance (Figs. S1 and S4).

Incomplete dominance was almost fully supported: egg masses presented broad ranges of egg tolerance with a unimodal distribution (Tables T3 and T4, Figs. S2 and S4). All possible unimodal tolerance patterns could be explained by incomplete dominance (up to 95% of the collected egg masses for AMD up to 97.5% for copper). A partially lethal pulse of AMD or copper would wipe out the most sensitive genotypes, although not fixing in the population the allele conferring tolerance. Allele’s fixation would happen only after exposure to an almost fully lethal concentration (only the homozygous tolerant genotypes would survive), depending on the degree of incomplete dominance, encompassing from almost full recessivity to almost full dominance. Nevertheless, neither of the alternative scenarios discussed above (full recessivity, full dominance, overdominance or underdominance) can be totally excluded. These less likely patterns could be supported by 38% of the egg masses exposed to AMD and by 22% of those exposed to copper. This 16% difference between the two contaminants can be due to the fact that tolerance to acid mine drainage may involve different physiological mechanisms than that to copper, probably because of the different pH, the former being notably acid while the latter rather neutral. Indeed, pH can influence metallic ions speciation (biotic-ligand-model theory)^40^. At least for fish (but arguably for others aquatic vertebrates breathing through gills), increasing pH decrease copper ions absorption^40^. However, because the acid mine drainage contains much more than copper alone^29^, its toxicity is possibly due also to other ions.

In all the possible patterns of tolerance inheritance, very tolerant and very sensitive egg masses should present a small relative spread: intermediately tolerant egg masses should present higher spread. Because the latter egg masses would comprise only very sensitive and very tolerant eggs, with the exception of the incomplete dominance pattern for which the latter egg masses include intermediately sensitive and tolerant eggs. Thus, when comparing the relationship between relative spread and LT_50_ values, the only expected shape for the distribution would be that of a crystal clear inverted-U. Which would be much less evident only if tolerance inheritance is due to incomplete dominance. This was found in the present study, for both AMD and copper (Figs. S3 and S5), further supporting incomplete dominance as the mechanism of tolerance inheritance.

A reasonable assumption is that each egg mass was fertilized by a one male. However, amplexi involving more than one male were reported for other amphibian species^41^. Some studies described multiple paternity in some anuran species, but the offspring of polyandrous mating were always a small proportion. In *Rana temporaria*, where high clutch piracy was reported (84% of clutches), the secondary males fertilized only 26% of the clutches and, in these clutches, only 24% of embryos were fertilized by pirate males^42^. Basically only 5%, or less, of all eggs collected at that breeding season were sired by a secondary male^42^. This is also the case in *Rana dalmatina*, in which only 4% of all eggs were sired by a secondary male^43^. It is reasonable that the effect and occurrence of polyandry, if present at all, would be negligible; however, secondary fertilization might result in egg masses with a possibly broader genetic makeup. Such situation could lead to an egg mass with a very large relative spread, which matches egg mass A and masses K14, Q14, S14, P16, and Q16 (Tables T3 and T4, Figs. S2 and S4). Those masses could also result from the simultaneous presence of extremely tolerant and sensitive eggs and the almost or full absence of intermediately tolerant eggs. This could also be determined by tolerance being a trait other than incompletely dominant or, at much less extent, arise from a polyandric mating^41^, especially in the cases of masses which show a bimodal distribution. However, as far as we are aware of, there are no evidences of multiple paternities in *P. perezi*. In the closest relatives for which information is available, the percentage of eggs sired by a secondary father is very low, with offspring of polyandrous mating being always a small proportion^42,43^. Furthermore, bimodality is clearly the exception rather than the rule in our data (Figs. S2 and S4).

The low occurrence of safely tolerant egg masses (S, T and U: only three out of 20 for AMD; O14, P14, R14, S14, R16, S16 and T16: only seven out of 40 for copper) compared with those critically sensitive (B, C, D, E, F, G, H: seven out of 20 for AMD; A14, B14, C14, D14, E14, F14, A16, C16, D16, E16: 17 out of 40 for copper), could be explained by the lack of past selection for tolerance to metal contamination.

## Conclusion

The above deductions were obtained according a genetic system of a single gene with two alleles. Such an assumption is plausible because previous studies reported that few major genes determine the tolerance to very intense selective pressure^5,6,27^. If, as it is true for some contaminants, survival to almost fully lethal concentrations of the tested stressors is ruled by dominant and recessive alleles, then the present results indicated a plausible inheritance pattern. However, other systems of genetic determination could be possible, for example a situation in which more than two alleles at a single gene would produce a gradient of tolerance, but incomplete dominance would still be the most probable mechanism; because this scenario is the one supported by the largest percentage among the tested egg masses. Even if egg tolerance to lethal levels of AMD and/or copper has a polygenic basis and even if the implicated genes have more than two alleles, incomplete dominance could remain valid for the trait as a whole at least at some genes, although the probability of losing alleles could theoretically be lower. In conclusion, the results seem to preliminarily support the recessive (or incompletely dominant) tolerance inheritance (working-) hypothesis^3^, highlighting the need to further address and prevent contaminant-driven genetic erosion, because of the possible irreversible loss of alleles (especially in small populations as those of amphibians). Combining heritability tests with genomics would be an auspicious future step.

## Supplementary information

Supplementary material for: Genetically inherited tolerance may unveil trait dominance patterns in an amphibian model.

## Data Availability

The authors declare to make materials, data and associated protocols promptly available to readers without undue qualifications in material transfer agreements.
