# Supplementary material for: Genetically inherited tolerance may unveil trait dominance patterns in an amphibian model
Source: Sci Rep. 2019 Dec 16;9:19179. doi: 10.1038/s41598-019-55838-9 (PMC6914805; doi:10.1038/s41598-019-55838-9)
Supplement: Supplementary file 1 — Supplementary material for: Genetically inherited tolerance may unveil trait dominance patterns in an amphibian model. [file 41598_2019_55838_MOESM1_ESM.pdf]

Supplementary material for:

Genetically inherited tolerance may unveil trait dominance patterns in an amphibian model.

E. Fasola[a,b,*] , R. Ribeiro[b], I. Lopes[a]

a Department of Biology & CESAM (Centro de Estudos do Ambiente e do Mar), University of Aveiro, Campus de Santiago, 3810-193 Aveiro, Portugal

b Department of Life Sciences & CFE (Centre for Functional Ecology), University of Coimbra, Calçada Martim de Freitas, 3000-456 Coimbra, Portugal

* Corresponding author: Emanuele Fasola; Department of Biology, University of Aveiro, Campus de Santiago, 3810-193 Aveiro, Portugal; +351 916707723; emanuele.fasola@ua.pt

Supplementary material for:

Genetically inherited tolerance may unveil trait dominance patterns in an amphibian model.

E. Fasola[a,b,]* , R. Ribeiro[b], I. Lopes[a]

a Department of Biology & CESAM (Centro de Estudos do Ambiente e do Mar), University of Aveiro, Campus de Santiago, 3810-193 Aveiro, Portugal

b Department of Life Sciences & CFE (Centre for Functional Ecology), University of Coimbra, Calçada Martim de Freitas, 3000-456 Coimbra, Portugal

* Corresponding author: Emanuele Fasola; Department of Biology, University of Aveiro, Campus de Santiago, 3810-193 Aveiro, Portugal; +351 916707723; emanuele.fasola@ua.pt

S1 Supplementary methods:

S1.1 Study organism:

The model organism for this study was the Iberian Water Frog: *Pelophylax perezi* (López-Seoane, 1885). The species has a conservation status of least concern by the International Union for the Conservation of Nature (1). It is endemic and common in the Iberian Peninsula and southern France. It was introduced in Madeira, Balearics, central as well as west Canary and Azores islands and in the United Kingdom (2). It can be encountered in rivers, streams and irrigation ditches, preferring areas with low current. It is very common in ponds, marshes, and reservoirs (2). It can tolerate temperatures up to 35°C and down to 3°C and it can be found at altitudes up to 2300 meters, in the Spanish Sierra Nevada mountains (2,3). The Iberian Water Frog can colonize eutrophic and even contaminated waters (4,5), making it a pioneer species in recovering habitats. This ability to cope with environmental stressors makes this species ideal to compare population responses between pristine and contaminated environments.

S1.2 Assay setup - AMD:

During seven visits, from 13th April 2013 to 8th August 2013, 21 *P. perezi* egg masses were collected at Quinta da Boa Vista (central west coast of Portugal, 40°35’48”N – 8°41’43”W), which is considered a non-contaminated reference site adjacent to the Natura 2000 site PTCON0061 (http://natura2000.eea.europa.eu/Natura2000/SDF.aspx?site=PTCON0061). Masses were only collected if they had been laid in the previous 24h, i.e. containing eggs at developmental Gosner's stages 8 to 10. Data on the average air temperature, the amount of rain, the average wind speed, and the average insulation at each day of egg collection were obtained from the datasets of the Portuguese environmental agency, Instituto Português do Mar e da Atmosfera (https://www.ipma.pt/pt/index.html). In the laboratory the masses were temporarily housed in small water aquaria (20x20x30 cm) containing Frog Embryo Teratogenesis Assay-Xenopus (FETAX) medium (6). Tests were performed in plastic Petri dishes (55 mm diameter) with 10 ml test solution. Each Petri dish contained 5 eggs. Eggs were exposed to a 60% dilution (with FETAX medium) of the

acid mine drainage (AMD) collected at the São Domingos mine (southeast Portugal, 37°39'15"N – 7°30'31"W). São Domingos mine was active from 1859 to 1965, is abandoned since; however, still highly impacts the surroundings due to the ongoing oxidation of mine tailings. This AMD is very acid (pH≈2), highly rich in metals, with an electrical conductivity around 4500 mS/cm (35). Metal concentrations were quantified in the 100% AMD and in a 10% dilution, by graphite furnace atomic absorption (Cd, Co, Ni, and Pb), by inductively coupled plasma atomic emission (Al, Cu, Fe, Mn, and Zn), and by hydride generation atomic absorption (As), in a certified laboratory (Instituto Superior Técnico, Lisbon, Portugal) (Table S1). Because metal concentrations in the 10% dilution are approximately one tenth of those in the 100% AMD (Table S1), metal concentrations in a 60% dilution are expected to be approximately six-fold higher than those in the 10% AMD dilution. The pH in the 60% dilution resulted very acid (pH≈3). Eggs from different egg masses were not mixed, i.e. each mass was analysed separately. For each egg mass, there were four control (FETAX medium) replicates (with five eggs each), in a total of 20 eggs; while the number of eggs exposed to the test solution varied depending on the mass size (usually between 16 and 20). The maximum number of exposed replicates per egg mass was 20 (100 eggs). Controls consisted of FETAX medium only. The eggs' jelly coat was not removed to mimic natural scenarios. Frog embryos were placed in a conditioned chamber (23°C) under a 14h/10h light/dark photoperiod. The exposure started the day of collection, lasting about 96h (details below). Embryos were checked for death (at a 10x magnification using a Leica MS5 microscope) following a predetermined base 10 logarithmic time scale (by successively adding 0.15 to the $\log_{10}720$): 12h00min (=720min), 16h57min, 23h57min, 33h49min, 47h46min, 67h29min, and 95h19min. The pH (Wissenschaftlich Technische Werkstätten pH330i, WTW, Weilheim, Germany) and conductivity (WTW conductivity440i) were measured following the same time schedule. During each assay, egg status and its development, response to gentle prodding and circulatory system functioning were sequentially inspected to assess embryos' death. The assay started with embryos approximately at Gosner's stage 9-10. Control eggs reached Gosner's stage 21-22 by the end of the experiments.

S1.3 Assay setup - Copper:

Sampling was performed from March 2014 to May 2014 and from March 2016 to April 2016. At each sampling season, 20 egg masses laid in the previous 24h (Gosner's stage 8-10) were collected, at Quinta de Boa Vista. The toxicity tests were performed as above. Except for the test solution, which was made by diluting with FETAX medium a stock solution (100 mg/L Cu) of $CuSO_4 \bullet 5H_2O$ (Merck, Kenilworth, NJ, USA), prepared with

with Milli-Q water (Academic System; Merck Millipore, Darmstadt, Germany), until a 9 mg/L of Cu concentration (resulting pH≈7). This value was chosen based on concentrations tested by Santos et al. (7) and was intended to be lethal within a 96h exposure period.

S1.4 Statistical analyses:

The following analyses were performed using Statistica for Windows 8.0 (StatSoft, Tulsa, OK, USA) and IBM SPSS for Windows v24 (IBM Corporation, Armonk, NY, USA). Values of median lethal time (LT_{50}) versus their respective relative spreads were checked for parametric correlation (Spearman test). Investigating the possible effects of environmental parameters on tolerance, correlations of rank of the collection day, average air temperature, amount of rain, average wind speed, average insulation at each day of egg collection versus the respective LT_{50} values were explored. Comparison between LT_{50} and spread values resulting from the two sampling seasons was performed conducting a Mann-Whitney U-test. Since inverted U-shaped relationships between relative spread and LT_{50} values were expected, the statistical significance of polynomial regression model fits was evaluated.

References:

1. Bosch, J., Tejedo, M., Beja, P., Martínez-Solano, I., Salvador, A., García-París, M. 2009. “Pelophylax Perezi. The IUCN Red List of Threatened Species.” http://dx.doi.org/10.2305/IUCN.UK.2009.RLTS.T58692A11812894.en.
2. Arnold, N., Ovenden, D. 2002. *Reptiles and Amphibians of Britain and Europe.* Collins; New edition.
3. Almeida, N, Almeida, P., Gonçalves, H., Sequeira, F., Teixeira, J., Almeida, F. 2001. *Guia FAPAS Dos Anfíbios e Répteis de Portugal.* Porto, Portugal: FAPAS.
4. Marques, S., Chaves, S., Gonçalves, F., Pereira, R. 2013. “Differential Gene Expression in Iberian Green Frogs (*Pelophylax Perezi*) Inhabiting a Deactivated Uranium Mine.” *Ecotoxicology and environmental safety* 87: 115–19. http://www.ncbi.nlm.nih.gov/pubmed/23146668 (November 7, 2013).
5. Sillero, N., Ribeiro, R. 2010. “Reproduction of Pelophylax Perezi in Brackish Water in Porto (Portugal).” *Herpetology Notes* 3: 337–340.
6. Organisation for Economic Co-operation and Development (OECD). 2008. “OECD Guidline for the Testing of Chemicals.” : 1–33.
7. Santos, B., Ribeiro, R., Domingues, I., Pereira, R., Soares, A., Lopes, I. 2013. “Salinity and Copper Interactive Effects on Iberian Water Frog Pelophylax Perezi.” *Environmental Toxicology and Chemistry* 32(8): 1864–72.

Figures:

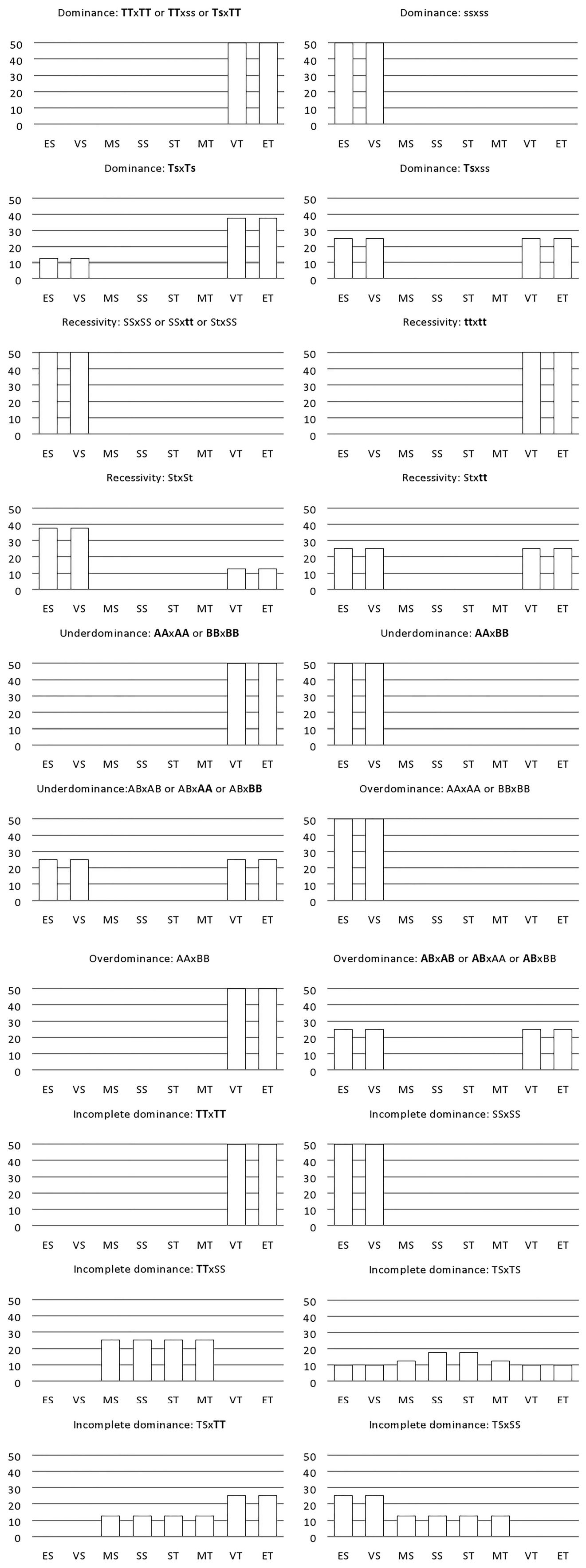
Dominance: TTxTT or TTxss or TsxTT
Dominance: ssxss
Dominance: TsxTs
Dominance: Tsxss
Recessivity: SSxSS or SSxtt or StxSS
Recessivity: ttxtt
Recessivity: StxSt
Recessivity: Stxtt
Underdominance: AAxAA or BBxBB
Underdominance: AAxBB
Underdominance:ABxAB or ABxAA or ABxBB
Overdominance: AAxAA or BBxBB
Overdominance: AAxBB
Overdominance: ABxAB or ABxAA or ABxBB
Incomplete dominance: TTxTT
Incomplete dominance: SSxSS
Incomplete dominance: TTxSS
Incomplete dominance: TSxTS
Incomplete dominance: TSxTT
Incomplete dominance: TSxSS
0 10 20 30 40 50
ES VS MS SS ST MT VT ET

Fig.S1: Expected theoretical frequencies (in %) of eggs (F1 generation), of Iberian Water Frog (*Pelophylax perezi*) egg masses, belonging to eight classes of lethal tolerance, in all possible scenarios of inheritance mechanism (dominance, recessivity, underdominance, overdominance, and incomplete dominance) from all possible parental genotype crossings in a two-alleles system (T and S) of a single gene. The classes correspond to the eight observation moments (following a logarithmic time scale from 12h00min up to 95h19min of exposure). T - allele conferring tolerance. S - allele not conferring tolerance. In the case of overdominance and underdominance (there is not an allele conferring tolerance), alleles were indicated as A and B. Lower case indicates the recessive allele. Bold face indicates the most tolerant genotypes. The X-axis represents time in subsequent observations. ES, VS, MS, SS, ST, MT, VT, and ET – Extremely, Very, Moderately, and Slightly Sensitive or Tolerant eggs, respectively.

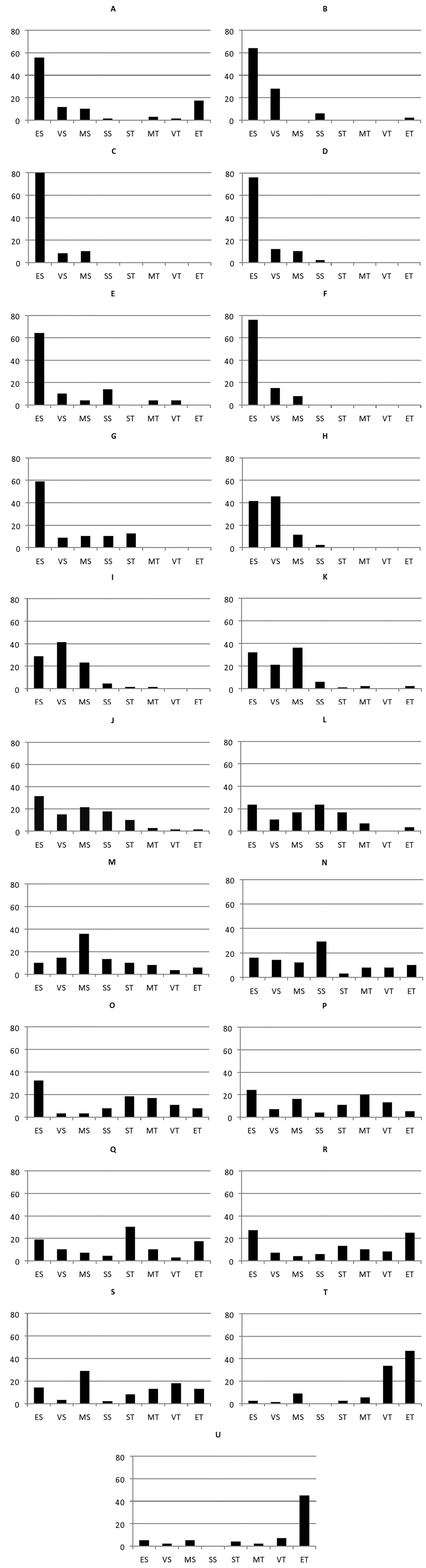
A
B
C
D
E
F
G
H
I
K
J
L
M
N
O
P
Q
R
S
T
U
0
20
40
60
80
ES VS MS SS ST MT VT ET

Fig.S2: Frequencies (in %) of eggs belonging to eight classes of lethal tolerance to a 60% dilution of acid mine drainage, in each of 21 Iberian Water Frog (*Pelophylax perezi*) egg masses (A to U), collected in a reference pond. The classes correspond to the eight observation moments (following a logarithmic time scale from 12h00min up to 95h19min of exposure). The X-axis represents time in subsequent observations. ES, VS, MS, SS, ST, MT, VT, and ET – Extremely, Very, Moderately, and Slightly Sensitive or Tolerant eggs, respectively.

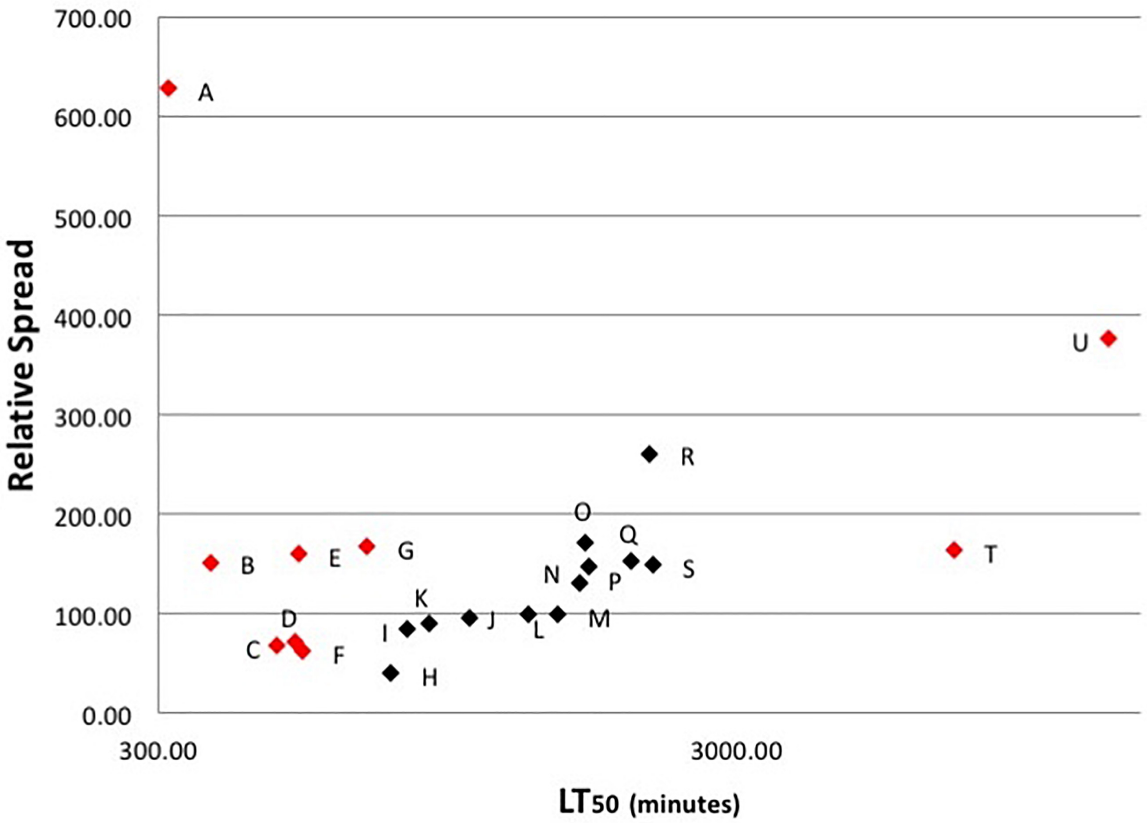

Fig.S3: Relative spread (difference between the upper and lower quartiles relatively to the median) of egg mass tolerance to a 60% dilution of metal-rich acid mine drainage of 21 Iberian Water Frog (*Pelophylax perezi*) egg masses, collected at a reference pond, versus the respective median lethal time values (LT_{50} – exposure time at which 50% of eggs were found dead). Red points indicate extrapolated LT_{50} values, i.e. being lower than the first observation (720 minutes) or higher than the last observation time (5719 minutes).

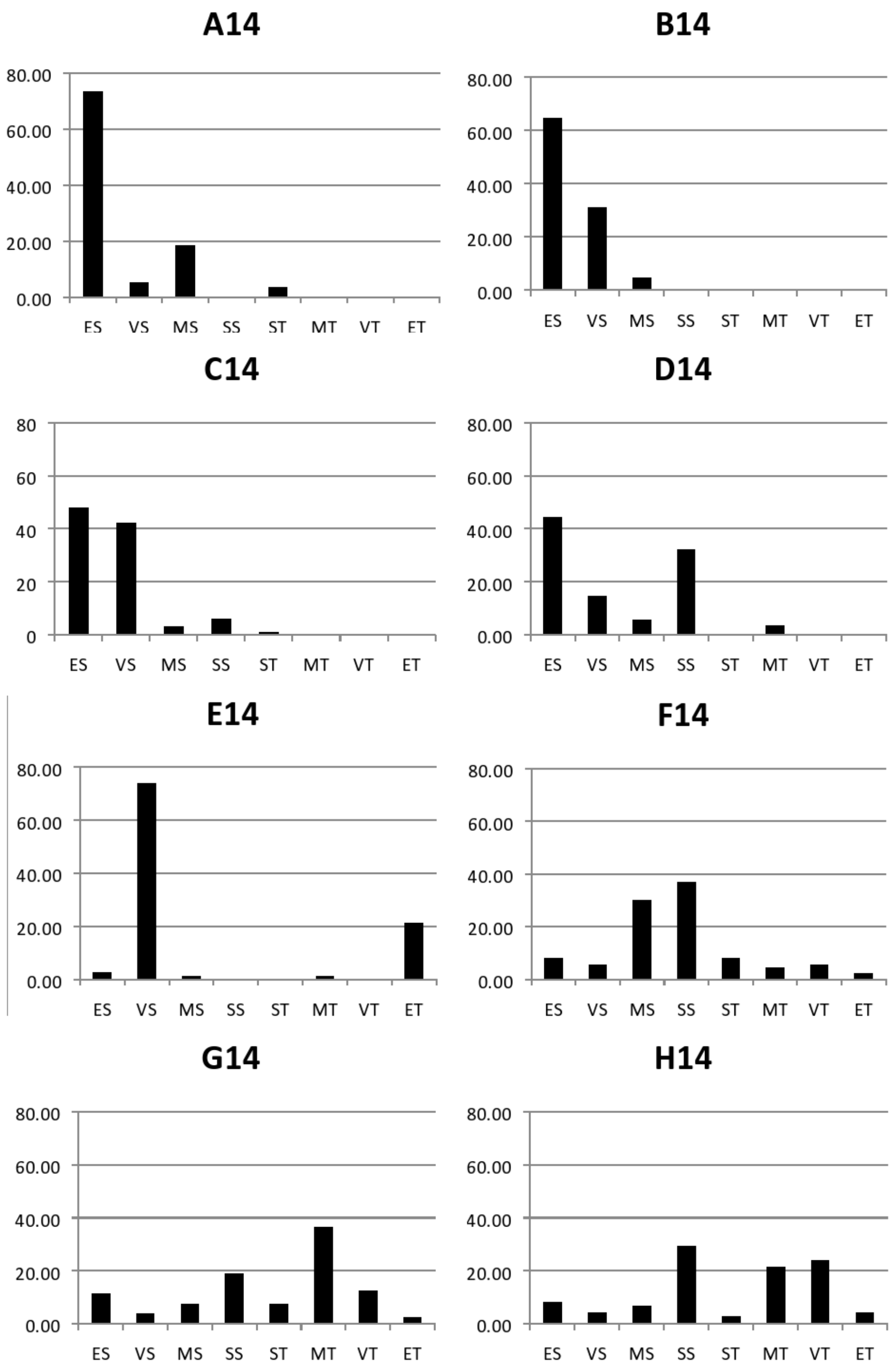
A14
B14
C14
D14
E14
F14
G14
H14
80.00
60.00
40.00
20.00
0.00
80
60
40
20
0
ES VS MS SS ST MT VT ET

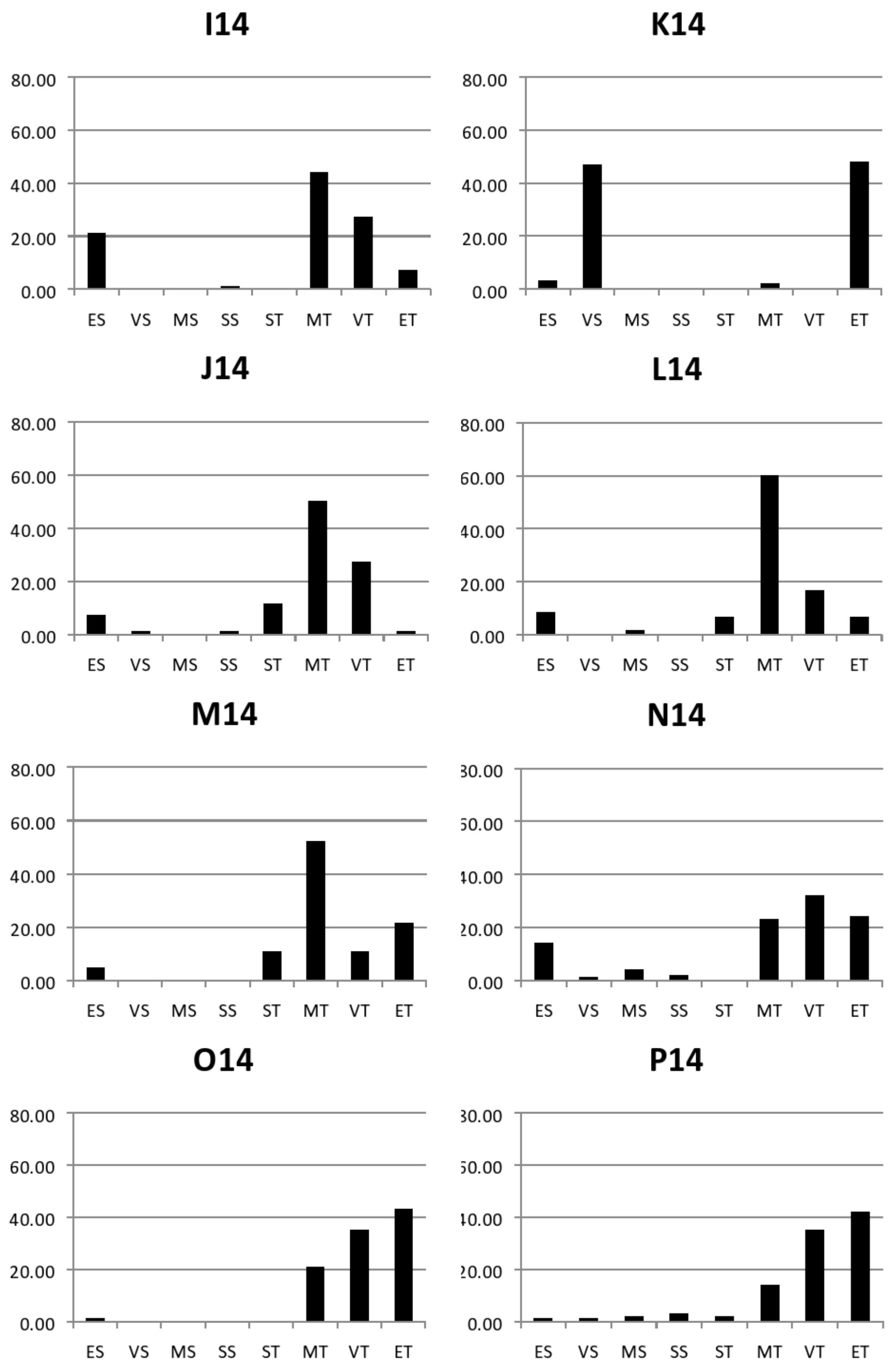
I14
K14
J14
L14
M14
N14
O14
P14
80.00
60.00
40.00
20.00
0.00
ES VS MS SS ST MT VT ET

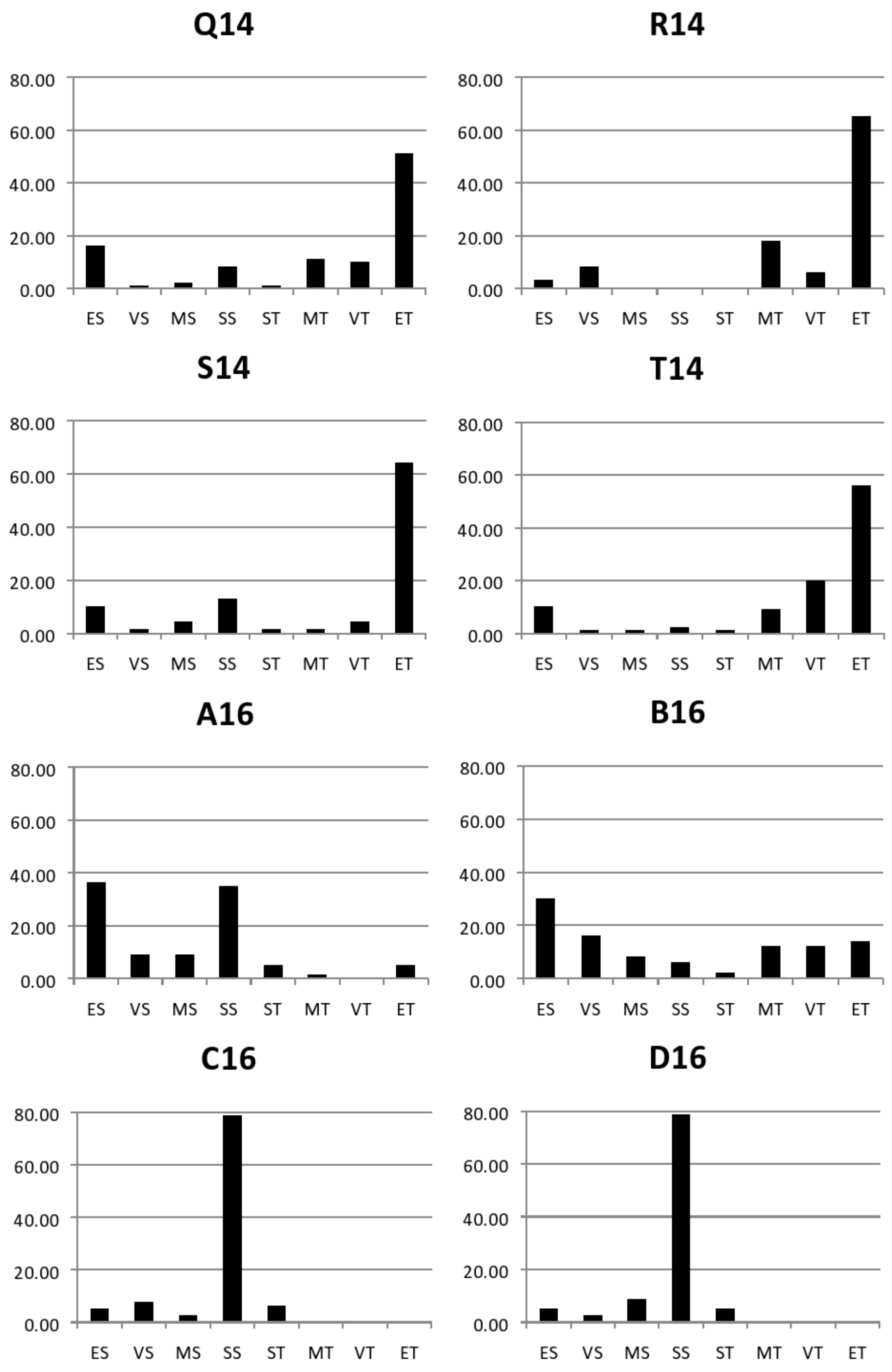
Q14
R14
S14
T14
A16
B16
C16
D16
80.00
60.00
40.00
20.00
0.00
ES VS MS SS ST MT VT ET

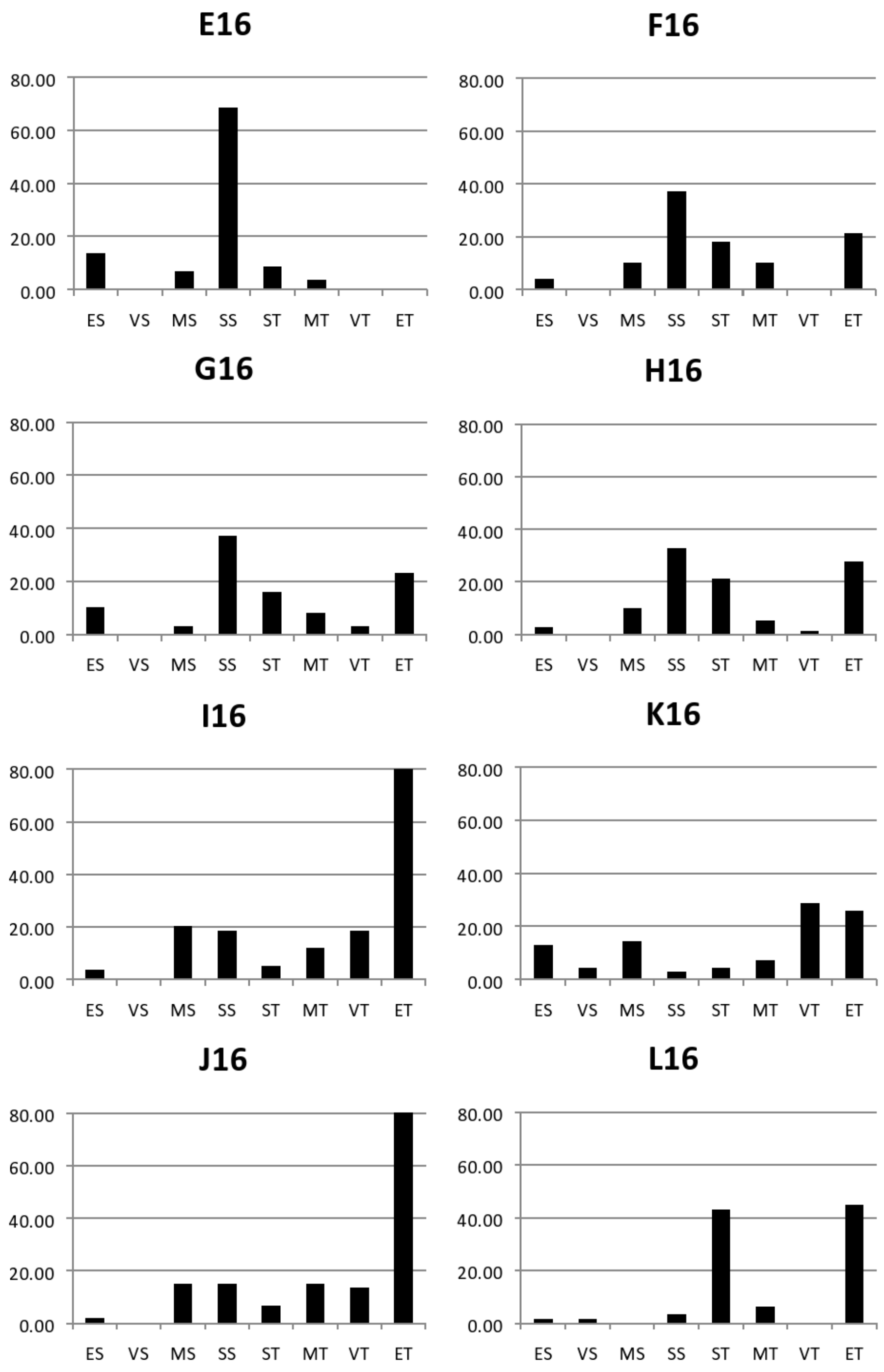
E16
F16
G16
H16
I16
K16
J16
L16
0.00
20.00
40.00
60.00
80.00
ES
VS
MS
SS
ST
MT
VT
ET

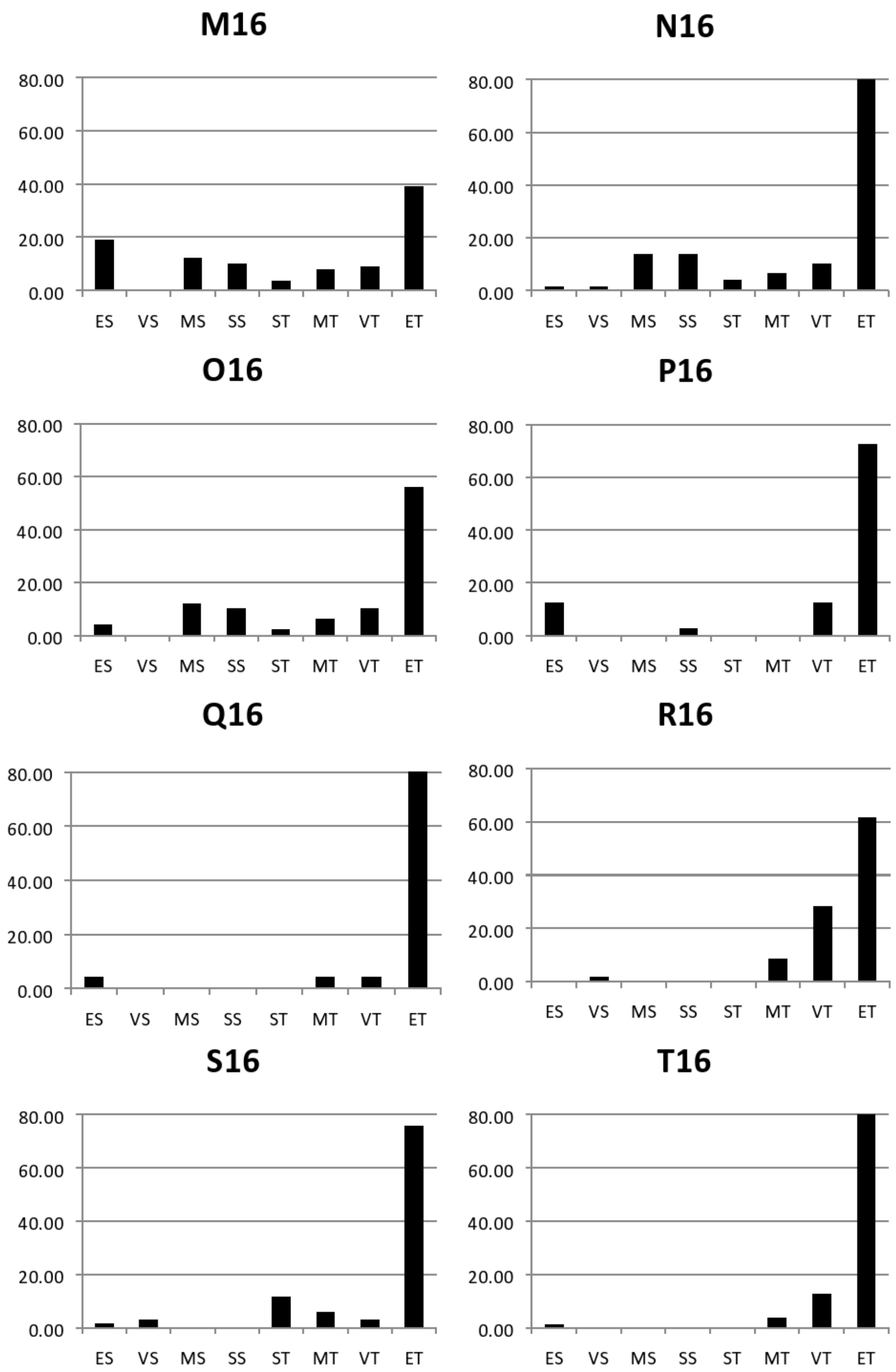

Fig.S4: Frequencies (in %) of eggs belonging to eight classes of lethal tolerance to 9mg/L of Copper, in each of 20 Iberian Water Frog (*Pelophylax perezi*) egg masses (A to T – 2014; a to t – 2016), collected in a reference pond. The classes correspond to the eight observation moments (following a logarithmic time scale from 12h00min up to 95h19min of exposure). The X-axis represents time in subsequent observations. ES, VS, MS, SS, ST, MT, VT, and ET – Extremely, Very, Moderately, and Slightly Sensitive or Tolerant eggs, respectively.

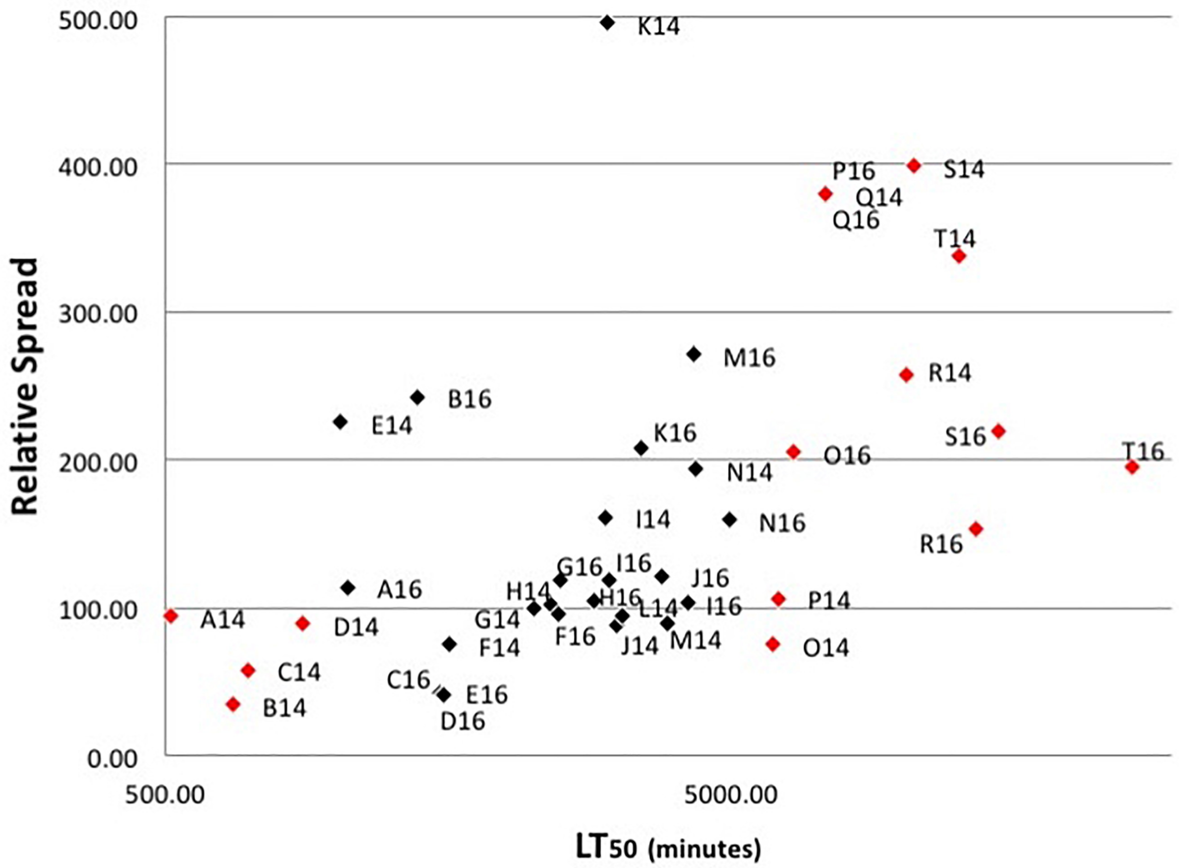

Fig.S5: Relative spread (difference between the upper and lower quartiles relatively to the median) of egg mass tolerance to 9 mg/L of copper for 40 Iberian Water Frog (*Pelophylax perezi*) egg masses, collected at a reference pond, versus the respective median lethal time values (LT_{50} – exposure time at which 50% of eggs were found dead). Red points indicate extrapolated LT_{50} values, i.e. being lower than the first observation (720 minutes) or higher than the last observation time (5719 minutes).

Tables:

Table T1: Metal concentrations (µg/L) in the acid mine drainage (AMD) from the São Domingos mine (southeast Portugal).

AMD	Al	Fe	Cu	Mn	Zn	Co	Ni	Cd	Pb	As
100%	$440x10^{3}$	$353x10^{3}$	$41x10^{3}$	$28x10^{3}$	$21x10^{3}$	$2.5x10^{3}$	800	308	24	1.5
10%	$46x10^{3}$	$33x10^{3}$	$4x10^{3}$	$2.9x10^{3}$	$2.1x10^{3}$	250	72	7.3	3	<1.0

Table T2: Median lethal time values (LT_{50}, minutes) with their 95% confidence limits and respective relative spread (%) of the 21 Iberian Water Frog egg masses exposed to a 60% dilution of the metal-rich acid mine drainage from the São Domingos mine (southeast Portugal). The average (minutes) of all LT_{50} values is also presented.

**Egg mass**	**LT_{50}**	**LT_{50} 95% conf. limits**	**Relative spread**
**A**	312.12	61.85 – 878.36	627.24
**B**	369.30	46.00 – 646.07	150.29
**C**	483.21	323.65 – 583.23	66.57
**D**	518.82	383.04 – 611.52	70.06
**E**	524,31	383.39 – 651.17	158.64
**F**	533.51	395.04 – 689.52	61.16
**G**	690.55	579,04 – 792.65	166.21
**H**	760.90	760.90 – 760.90	39.00
**I**	809.99	572.32 – 1226.08	82.55
**K**	883.14	1309,58 – 2002.27	89.00
**J**	1034.86	936.07 – 1130.22	95.33
**L**	1309.58	1200.23 – 1417.85	97.96
**M**	1475.26	1241.11 – 1712.82	97.40
**N**	1615.01	1457.44 – 1751.36	129.83
**O**	1648.43	1166.15 – 2093.03	170.96
**P**	1670.49	1309.58 – 2002.27	146.54
**Q**	1972.70	1533.19 – 2336.57	152.05
**R**	2129.63	1645.71 – 2566 52	259.86
**S**	2151.56	1645.71 – 2566.52	147,99
**T**	7155.24	3281.15 – 9112.73	162.87
**U**	13239.40	2962.91 – 22109.80	375.08

Mean LT_{50} (minutes)
1966.10

Table T3: Median lethal time values (LT_{50}, minutes) with their 95% confidence limits and respective relative spread (%) of the 20 Iberian Water Frog egg masses exposed to 9 mg/L of copper (year 2014). The average (minutes) of all LT_{50} values is also presented.

**Egg Mass**	**LT_{50} (minutes)**	**LT_{50} 95% conf. limits**	**Rel. Spread**
**A14**	510.32	377.34 - 613.14	94.40
**B14**	656.68	585.52 - 702.56	34.20
**C14**	697.37	617.35 - 760.68	57.60
**D14**	874.14	603.20 - 1080.81	88.79
**E14**	1018.78	805.76 - 1368.39	225.26
**F14**	1582.22	1339.84 -1844.95	75.19
**G14**	2230.76	1767.15 -2879.11	99.92
**H14**	2389.03	1983.21 - 2931.47	101.61
**I14**	2989.79	1580.31 - 4500.63	160.96
**K14**	3016.58	1865.46 - 4751.32	495.47
**J14**	3142.71	1960.32 - 8661.77	87.96
**L14**	3211.62	1948.36 - 10923.40	94.28
**M14**	3856.35	2557.67 - 10774.75	89.54
**N14**	4326.76	2755.45 - 17824.22	193.60
**O14**	5904.50	3295.35 - 19827.46	75.04
**P14**	6025.90	4597.20 - 10551.57	106.03
**Q14**	7320.20	5233.49 - 13145.48	379.90
**R14**	10185.10	5760.08 - 16929.11	257.35
**S14**	10491.40	6844.12 - 23548.46	398.92
**T14**	12578.20	6216.81 - 169212.03	337.74

Mean LT_{50} (minutes)

4150.42

Table T4: Median lethal time values (LT_{50}, minutes) with their 95% confidence limits and respective relative spread (%) of the 20 Iberian Water Frog egg masses exposed to 9 mg/L of copper (year 2016). The average (minutes) of all LT_{50} values is also presented.

Egg Mass	LT_{50} (minutes)	LT_{50} 95% conf. limits	Rel. Spread
**A16**	1045.72	696.59 - 1337.77	114.03
**B16**	1393.14	1157.02 - 1622.63	242.89
**C16**	1525.32	1143.42 - 2151.83	41.52
**D16**	1553.49	1273.12 - 2352.80	40.39
**E16**	1555.91	1279.27 - 2452.87	40.39
**F16**	2477.99	1933.65 - 3249.81	95.48
**G16**	2496.92	1944.36 - 3334.01	118.45
**H16**	2868.43	2201.98 - 4001.46	104.40
**I16**	3035.57	2526.89 - 3814.64	118.92
**K16**	3454.40	1865.46 - 4751.32	208.13
**J16**	3761.61	2981.14 - 4157.42	121,64
**L16**	4180.08	3087.37 - 7651.67	103.20
**M16**	4272.60	3505.26 - 5654.01	271.42
**N16**	4940.60	3683.04 - 8593.13	159.15
**O16**	6434.97	5168.01 - 8887.32	204.90
**P16**	7320.20	5233.49 - 13145.40	379.90
**Q16**	7321.29	5239.90 - 13148.48	379.90
**R16**	13483.70	6700.64 - 32251.20	153.02
**S16**	14798.90	9717.50 -31683.00	219.93
**T16**	25515.50	13703.41 -106548.21	195.31
		**Mean LT_{50} (minutes)**	
		5671.64	
